# Polygenic risk of Parkinson disease is correlated with disease age at onset

**DOI:** 10.1002/ana.24335

**Published:** 2015-03-13

**Authors:** Valentina Escott‐Price, for the International Parkinson's Disease Genomics Consortium, Mike A. Nalls, Huw R. Morris, Steven Lubbe, Alexis Brice, Thomas Gasser, Peter Heutink, Nicholas W. Wood, John Hardy, Andrew B. Singleton, Nigel M. Williams, on behalf of the IPDGC consortium members

**Affiliations:** ^1^ Institute of Psychological Medicine and Clinical Neurosciences, Medical Research Council Centre for Neuropsychiatric Genetics and Genomics, Department of Psychological Medicine and NeurologyCardiff University School of MedicineCardiffUnited Kingdom; ^2^ Laboratory of Neurogenetics, National Institute on AgingNational Institutes of HealthBethesdaMD; ^3^ Department of Clinical Neuroscience, Institute of NeurologyUniversity College LondonLondonUnited Kingdom; ^4^ Department of GeneticsParisFrance; ^5^ Cnrs, UMR 7225, ICM, F-75013ParisFrance; ^6^ Department for Neurodegenerative Diseases, Hertie Institute for Clinical Brain ResearchUniversity of TübingenTübingenGermany; ^7^ German Center for Neurodegenerative Diseases (DZNE)TübingenGermany; ^8^ University College London Genetics InstituteUniversity College LondonLondonUnited Kingdom

## Abstract

**Objective:**

We have investigated the polygenic architecture of Parkinson disease (PD) and have also explored the potential relationship between an individual's polygenic risk score and their disease age at onset.

**Methods:**

This study used genotypic data from 4,294 cases and 10,340 controls obtained from the meta‐analysis of PD genome‐wide association studies. Polygenic score analysis was performed as previously described by the International Schizophrenia Consortium, testing whether the polygenic score alleles identified in 1 association study were significantly enriched in the cases relative to the controls of 3 independent studies. Linear regression was used to investigate the relationship between an individual's polygenic score for PD risk alleles and disease age at onset.

**Results:**

Our polygenic score analysis has identified significant evidence for a polygenic component enriched in the cases of each of 3 independent PD genome‐wide association cohorts (minimum *p* = 3.76 × 10^−6^). Further analysis identified compelling evidence that the average polygenic score in patients with an early disease age at onset was significantly higher than in those with a late age at onset (*p* = 0.00014).

**Interpretation:**

This provides strong support for a large polygenic contribution to the overall heritable risk of PD and also suggests that early onset forms of the illness are not exclusively caused by highly penetrant Mendelian mutations, but can also be contributed to by an accumulation of common polygenic alleles with relatively low effect sizes. Ann Neurol 2015;77:582–591

The identification of rare highly penetrant mutations in genes causing familial and early onset Parkinson disease (PD)^1^, ^2^, ^3^, ^4^, ^5^ has considerably improved our understanding of disease pathogenesis. Recently, our understanding of idiopathic PD has been enhanced by genome‐wide association (GWA) studies^6^, ^7^, ^8^, ^9^, ^10^, ^11^, ^12^, ^13^, ^14^, ^15^, ^16^ that have collectively identified PD risk variants at >18 loci.^6^, ^7^ Despite their high levels of significance, these 18 loci are thought to account for only a very small amount (3–5%) of the expected heritability of PD.^17^ GWA study data sets can be used to determine a polygenic contribution of common single nucleotide polymorphisms (SNPs) that show disease association but fail to meet the significance threshold for genome‐wide significance (*p* > 5 × 10^−8^) to PD. Recent studies confirm that when weak effect loci are also considered^17^ there is a substantial increase in the estimated heritability detected in PD GWA studies (24%), and this strongly implies that a large proportion of genetic signal must lie below the genome‐wide significance thresholds set in the primary analyses.

Polygenic score analysis tests whether the alleles of small effect that GWA studies are underpowered to detect confer an aggregate risk and whether the same sets of risk alleles are shared between cohorts/data sets.^18^ We have investigated the polygenic contribution to PD by assessing whether score alleles identified in a GWA study from the United Kingdom are significantly enriched in cases from 3 independent GWA studies.

The age at onset (AAO) of PD has a relatively high heritability^19^ and has been previously shown to be associated with a small number of common variants,^14^, ^20^ some of which are strongly associated with disease.^14^ Using polygenic score analysis, we have considered PD according to a liability threshold model. Conceptually, liability is a quantitative measure that represents all risk factors that determine whether an individual will develop a disease. The liability threshold model assumes that individuals with a total liability greater than or equal to a fixed threshold will develop the disease. Mendelian forms of PD are caused by rare highly penetrant mutations at a single disease locus and are also typically associated with a young AAO. In line with a liability threshold model, a rare highly penetrant mutation is likely to contribute to a large proportion of an individual's disease liability. We propose that if an individual's common risk allele polygenic score is related to their disease liability we might expect patients carrying the highest load of common risk alleles to develop PD at the youngest ages. Conversely, if a large proportion of these cases are monogenic in nature, one might expect an attenuation of any relationship between polygenic score and AAO in the very young, supporting the notion that early onset PD has a substantial monogenic component.^21^, ^22^, ^23^

Our study provides compelling evidence for a polygenic contribution to PD, and that an individual's polygenic score is correlated with age at disease onset. Importantly, this indicates that a liability threshold model is relevant to PD pathogenesis and that early onset forms of the illness are not limited to Mendelian subtypes.

## Subjects and Methods

### PD GWA Data Set

This study used data obtained from the meta‐analysis of 5 PD GWA studies (5,333 PD cases and 12,298 controls) of which 259,577 SNPs passed study‐specific quality controls in all studies.^6^ The summary statistics for each marker in the PD data set were obtained using fixed effect inverse variance weighted meta‐analyses with METAL software (http://www.sph.umich.edu/csg/abecasis/metal/). The AAO and individual genotypes were available for a total of 4,111 PD cases. For these patients, AAO was systematically determined at the time of inclusion by a retrospective interview and is defined as the age at which PD was first diagnosed.

### Polygenic Score Analysis

We followed the approach previously described by the International Schizophrenia Consortium.^18^ Essentially this involved the selection of a set of SNPs that were in relative linkage equilibrium (*r* ^2^ < 0.25) and the generation of additive polygenic risk scores using SNPs with increasingly liberal probability values in a GWA study discovery data set, which were then tested for enrichment within an independent test sample.

In this study, we first investigated whether the polygenic score that was based on the PD GWA results of 1 data set were significantly enriched in the cases relative to the controls of another independent PD GWA study. For this analysis, we used 4 natural subsets of the International Parkinson's Disease Genomics Consortium (IPDGC) data where the individual genotypes of both cases (n = 4,294) and controls (n = 10,340) were available. After random pruning for linkage disequilibrium (LD; *r* ^2^ < 0.25), there were 59,770 SNPs available for polygenic score analyses. We used our most powerful PD case/control subset as our discovery sample (UK GWA study; 1,705 PD cases/6,200 controls) to select SNPs associated with PD, for each identifying its probability value for tests of allelic association, the effect size, and the allele that was present in the PD group more frequently than in the controls. We termed these the "score" alleles and further categorized them according to whether they met a predetermined significance threshold of association (*p* < 0.01, 0.05, 0.1, up to 0.5). We next used PLINK v1.07 to calculate the polygenic score for each individual in each of 3 independent case/control cohorts (USA‐1, 876 cases/859 controls; USA‐2, 971 cases/937 controls; Germany‐1; 742 cases/667 controls) as the average number of score alleles they possessed, each weighted by their effect size (B‐coefficient) log of odds ratio (OR) from the PD discovery sample. Logistic regression was then used to test whether the polygenic score distinguished case/control status in the 3 independent studies (USA‐1, USA‐2, and Germany‐1).

### Age at Onset Analysis

To maximize the quality of the LD pruning, we considered it worthwhile to use the largest and most powerful PD GWA study available to us (5,333 cases and 12,298 controls). LD pruning (using *r* ^2^ > 0.25, a physical distance threshold for clumping SNPs of 250kb, and *p* = 1 as the significance threshold for SNPs, which allowed us to capture all SNPs, even if their association with PD was not significant) identified a set of 104,830 independent SNPs that retained those most significantly associated with the disease.

The LD‐pruned set of SNPs were subsequently used for polygenic score analysis in the 4,294 PD patients for whom genotype data were available (UK, German, USA‐1, and USA‐2 GWA studies). Of these, AAO data were available in 4,111 patients (mean AAO = 60.9 years, standard deviation = 12.6), for whom we identified the score alleles and calculated their polygenic score. Polygenic scores were then adjusted for the country of origin using linear regression, and the residuals were then normalized by subtracting the mean and dividing by the standard deviation.

To investigate the relationship between an individual's polygenic score for PD risk alleles and disease AAO, we initially used linear regression analyses of the entire data set. Because clinical estimates of the AAO of PD will inevitably have limited precision in predicting the start of an individual's underlying biological pathology, we also used chi‐square or, where appropriate, Fisher exact tests to compare the polygenic score between individuals with AAO at the extremes ends of the AAO distribution.

## Results

### Polygenic Risk Score Analysis

In this study, we investigated whether the polygenic score alleles identified in 1 PD GWA study were significantly enriched in the cases relative to the controls of independent PD data sets. Polygenic score analysis revealed significant evidence for an overall enrichment of the PD score alleles identified in the UK GWA discovery sample^14^ in the cases of each of 3 independent PD GWA cohorts from the USA (×2) and Germany (Table 1).

**Table 1:** Results of Polygenic Score Analysis of PD Score Alleles in 3 Independent PD Target Samples

Selection Threshold of Score SNPs in UK Discovery Sample	No. of Significant SNPs at *p*_T_ in UK Discovery Sample	*p* for Polygenic Score Association in Each Target Sample		
		Germany‐1	USA‐1	USA‐2
*p*_T_ < 0.0001	9	0.822	0.039	0.117
*p*_T_ < 0.001	64	0.711	0.782	0.014
*p*_T_ < 0.01	655	0.992	0.064	0.156
*p*_T_ < 0.05	3,167	0.976	0.067	0.004
*p*_T_ < 0.1	6,265	0.160	0.009	0.003
*p*_T_ < 0.2	12,256	2.56 × 10^−3^	1.9 × 10^−3^	4.1 × 10^−5^
*p*_T_ < 0.3	18,281	2.52 × 10^−4^	3.44 × 10^−4^	2.65 × 10^−5^
*p*_T_ < 0.4	24,169	2.17 × 10^−4^	1.94 × 10^−4^	3.76 × 10^−6^
*p*_T_ < 0.5	30,157	8.22 × 10^−5^	4.42 × 10^−4^	1.83 × 10^−5^

PD = Parkinson disease; *p*_T_ = significance level probability value thresholds for SNP selection in the discovery sample; SNP = single nucleotide polymorphism.

In accordance with the pattern seen in studies of other complex diseases shown to have a polygenic signal,^18^, ^24^, ^25^, ^26^ restricting our analysis to SNPs that met the lowest association test probability value thresholds (*p*_T_) in the discovery sample (*p*_T_ < 10^−4^, *p*_T_ < 10^−3^, *p*_T_ < 0.01, *p*_T_ < 0.05) did not identify a systematic significant inflation in the polygenic scores of the PD cases of the replication samples (*p* > 0.05). Rather, our most significant evidence was observed when SNPs with *p*_T_ ≤ 0.5 in the UK sample were included where probability values for a significant inflation in the polygenic scores ranged between 4.42 × 10^−4^ and 8.22 × 10^−5^ (see Table 1). For all significant associations the B‐coefficients were positive, indicating that the higher polygenic score in the UK discovery sample corresponds to the higher score in each of the 3 independent replication samples and provides evidence for a polygenic contribution to the development of PD.

### Polygenic Score and AAO

To investigate a potential relationship between an individual's polygenic score and their AAO, we initially used linear regression of all 4,111 PD patients. This revealed nominally significant evidence that AAO was correlated with polygenic score but only when the analysis was restricted to SNPs with *p*_T_ < 0.01 (Table 2). Closer inspection of the regression analyses revealed that although falling short of nominal significance the B‐coefficients were negative at all *p*_T_ cutoffs, indicating that our data showed a consistent trend of higher polygenic score corresponding to an earlier AAO of PD.

**Table 2:** Linear Regression Analysis for Association of the Polygenic Score with Age at Onset

*p*_T_	No. of Significant SNPs at *p*_T_	Effect (B‐coefficient)	*p*	*R* ^2^
1.00E‐04	90	−0.48	0.0135	0.0015
0.001	528	−0.50	0.0103	0.0016
0.01	3,252	−0.43	0.0304	0.0011
0.05	11,757	−0.31	0.1172	0.0006
0.1	20,691	−0.32	0.1072	0.0006
0.2	35,655	−0.32	0.1003	0.0007
0.3	48,448	−0.31	0.1163	0.0006
0.4	59,852	−0.34	0.0830	0.0007
0.5	69,666	−0.33	0.0917	0.0007

*p*_T_ = significance level probability value thresholds for SNP selection; SNP = single nucleotide polymorphism.

**Figure 1:**
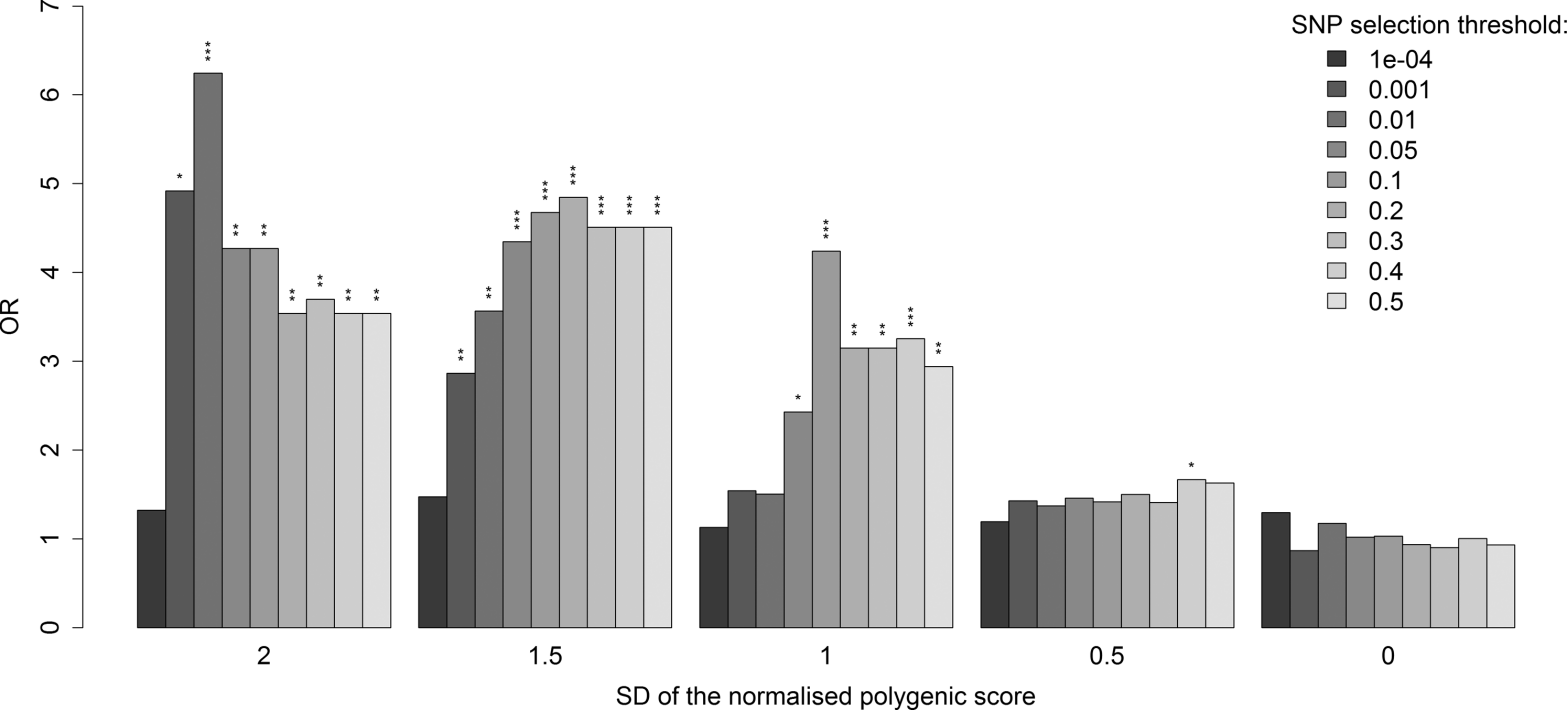
Assessment of the level that polygenic scores > 0 are enriched in Parkinson disease patients with age at onset (AAO) < 40 years compared to AAO > 80 years. *p < 0.05, **p < 0.01, ***p < 0.001. OR = odds ratio; SD = standard deviation; SNP = single nucleotide polymorphism.

We recognize that imprecision in the clinical estimates of the AAO of PD could adversely affect the power of our regression analysis. We therefore next compared the polygenic scores of patients whose AAO was at the lower 5% (AAO < 40 years, n = 248) with those at the upper 5% (AAO ≥ 80 years, n = 196) of the AAO distribution. This revealed that patients with an AAO < 40 years had a significantly higher polygenic score (mean = 0.14, standard error [SE] = 0.076) than those with an AAO ≥ 80 years (mean = −0.05, SE = 0.059). Looking at the numbers of patients with higher polygenic score categorized by early versus late onset, we consistently observed this pattern (OR > 1) at all *p*_T_ values and for all polygenic scores cutoffs > 0 (Fig 1). Our most significant result was when we compared patients with polygenic scores > 1.5 (*p*_T_ = 0.2, *p* = 0.00014), which revealed that 33 (13%) of patients with a polygenic score > 1.5 had an AAO < 40 years, whereas only 6 (3%) had an AAO ≥ 80 years (OR = 4.8, relative risk [RR] = 4.3). Moreover, our data also revealed a consistent relationship between disease AAO and polygenic score at all *p*_T_ thresholds.

**Figure 2:**
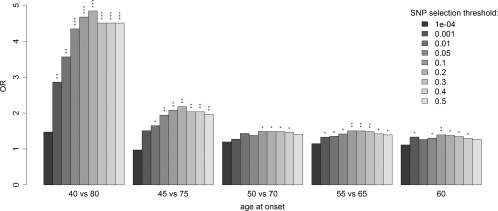
Assessment of the level that polygenic scores > 1.5 are enriched in different thresholds of early versus late onset Parkinson disease patients. *p < 0.05, **p < 0.01, ***p < 0.001. OR = odds ratio; SNP = single nucleotide polymorphism.

Relaxing our AAO threshold by ±5 years at either end of the AAO distribution demonstrated that although we consistently observed the same pattern at all thresholds of AAO, our strongest effects were seen when comparing the patients at the most extreme ends of the AAO distribution (Fig 2). It has previously been reported that the genetic structure in a population can be correlated with age.^27^ We investigated the possibility of this adversely affecting our results by performing an analogous analysis that compared the distribution of the PD score alleles between the oldest and youngest 5% (corresponding to <50 years and >87 years, respectively) of an independent cohort of Alzheimer disease (AD) patients (3,177 AD cases and 7,277 controls).^28^ This failed to identify a significant difference in the distribution of the PD score alleles between the 2 age groups (minimum *p* = 0.49, data not presented). We therefore conclude that the most likely explanation for our results being strongest when comparing PD patients at the most extreme ends of the AAO distribution is a reduction in power due to the inherent imprecision of relating age at diagnosis to a biological AAO and also that our selection of PD score alleles inevitably captures a proportion of SNPs that are not causal.

**Figure 3:**
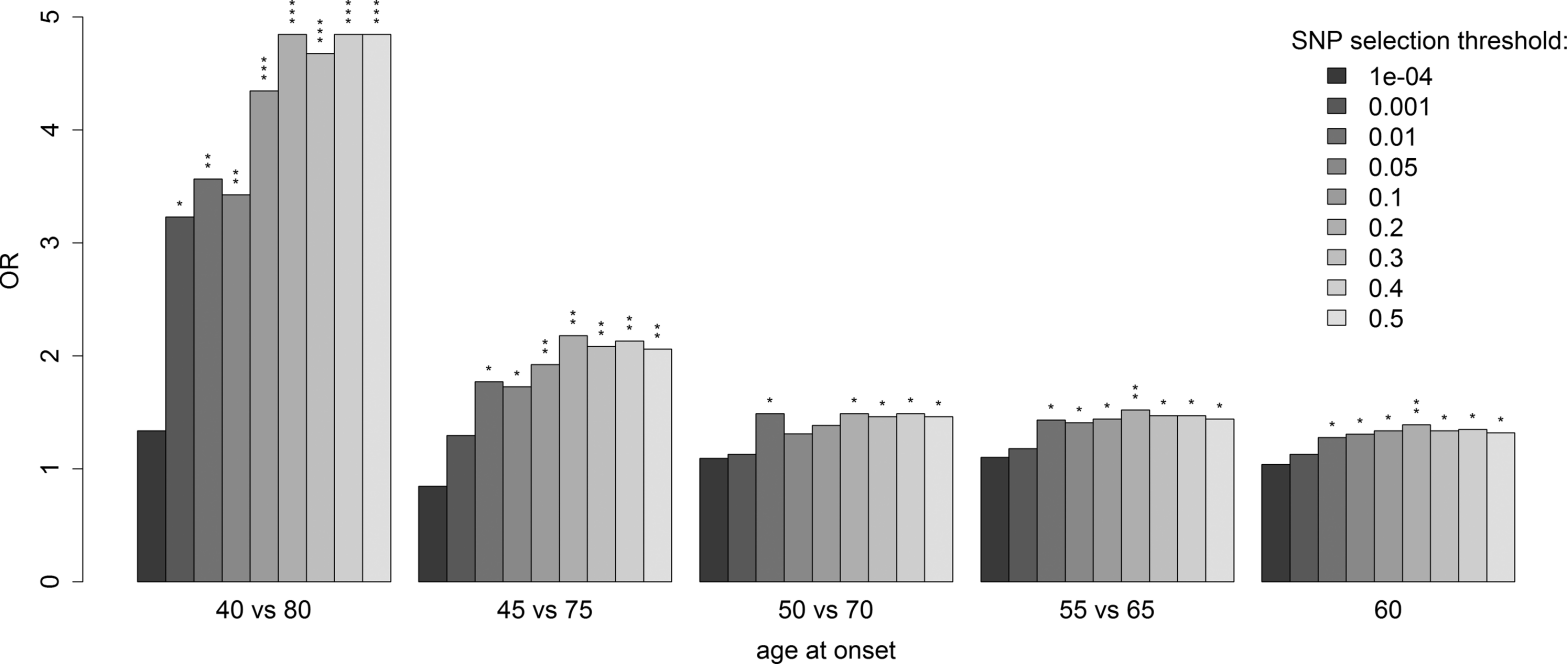
Assessment of the level that polygenic scores > 1.5 are enriched in different thresholds of early versus late onset Parkinson disease (PD) patients when 18 regions reported to be associated with PD are excluded. *p < 0.05, **p < 0.01, ***p < 0.001. OR = odds ratio; SNP = single nucleotide polymorphism.

Because the 104,830 independent SNPs that we used to identify PD score alleles included those most significantly associated with the disease, it is plausible that our results are being artificially biased by SNPs whose evidence for association is due to or merely a consequence of LD with the very strong association signal of known GWA study hits. To investigate this possibility, we repeated our analysis using identical analysis thresholds but this time excluding all 1,729 SNPs that after LD pruning were present at the 18 genomic regions previously reported to be strongly associated with PD, including the human leukocyte antigen locus.^6^ Given that each of these regions is likely to span at least 1 true PD susceptibility allele that would now be excluded from our polygenic score analysis, this approach is highly conservative. Nevertheless, this analysis again revealed significant evidence that individuals with higher polygenic scores had on average a lower AAO of PD, with our most significant result indicating the same magnitude of RR and OR between polygenic score and AAO (Fig 3). Moreover, we also obtained analogous results when we used an alternative method of LD pruning that ignored the strength to which SNPs were associated with PD and also excluded SNPs from the 18 associated regions (data not presented). These analyses suggest that our findings are not dependent on either the previously identified susceptibility loci or the SNPs that are falsely associated with PD merely as a consequence of LD with the very strong association signals.

## Discussion

The molecular genetic data reported in this study provides strong support for a large polygenic contribution to the overall heritable risk of PD. This implies that the genetic architecture of PD includes many common variants of small effect and is likely to be reflected in a large number of susceptibility genes and a complex set of biological pathways relevant to the disease. The PD score alleles identified in this cohort are not significantly enriched (minimum *p* = 0.14) in an independent GWA study for AD,^29^ indicating that the polygenic component to PD that we have identified is disease specific. Moreover, to conduct our analysis we had to define a training GWA study and a series of replication data sets. We achieved this by splitting the IPDGC meta‐analysis by its original GWA studies and observed a similar pattern of results when we defined the PD score alleles in a sample from the United Kingdom and tested for enrichment in samples from Germany and the USA (and vice versa). It is therefore unlikely that our observations are an artifact of subtle population substructure present in 1 of our sample cohorts.

We have also investigated the potential relationship between AAO and polygenic score. To do this we hypothesized that if a person's polygenic score represents a measure of their overall load of PD risk alleles then, in accordance with a liability threshold model, an individual's disease liability should be related to their polygenic score. For established PD risk factors, it is recognized that rare highly penetrant Mendelian variants (eg, homozygous *PARK2* mutations) typically lead to a reduced AAO when compared to less penetrant disease mutations (eg, G2019S at *LRRK2*). As Mendelian mutations are expected to represent a substantial proportion of a carrier's disease liability, we predicted that PD patients who do not carry highly penetrant Mendelian risk mutations but manifest the disease at a younger age would on average carry the highest polygenic load of common PD susceptibility alleles. Our study has identified compelling evidence that supports this hypothesis; patients with an early AAO consistently had a significantly higher polygenic score when compared to those with a late AAO. This indicates that early onset forms of PD are not limited to Mendelian genetic subtypes but can also be contributed to by an accumulation of common polygenic alleles. Moreover, as our study did not include a prescreening to identify highly penetrant mutations, it is possible that a small number of carriers remain in this cohort; our analysis can therefore be considered conservative.

As might be expected, we observed our strongest enrichment when we compared patients with an AAO at the lower and upper 5% of our sample distribution (OR = 4.8, RR = 4.3), which suggested that a PD patient with a polygenic score > 1.5 is ∼4 times more likely to develop the disease before the age of 40 years than after 80 years of age. Importantly, by adjusting the polygenic scores for the country of origin, we minimized any possible adverse effects of population stratification. Excluding all SNPs spanning genomic regions that harbor known PD susceptibility loci did not adversely affect our findings, implying that the main contribution of the PD polygenic signal identified in this study is from common SNPs that show disease association but fail to meet the probability value threshold for genome‐wide significance.

Further studies are required if we are to progress from evidence for a polygenic contribution to PD to understanding the specific genetic factors that comprise the polygenic component. Increasing the discovery sample size will allow more loci with increasingly small individual effect sizes to pass the threshold of genome‐wide significance, and should substantially refine the polygenic scores derived here. Moreover, as we have previously shown, using approaches such as gene pathway analyses it is possible to utilize the polygenic signal captured and identify genes or biological systems relevant to PD.^30^

It is possible that our findings are being influenced by rare PD susceptibility variants that are in LD with the common alleles analyzed in this study. The ongoing efforts of studies performing exome and whole genome sequencing in large numbers of PD case/control cohorts will allow us to establish the haplotype structure of common and rare alleles, and will allow us to understand which loci are subject to "synthetic association."^31^ Moreover, as previously demonstrated in other complex diseases,^32^ future polygenic score analysis of variants identified by exome/genome sequencing is expected to further inform our understanding of the genetic underpinnings of PD. Although it is an important measure, we recognize that clinical estimates of the AAO of PD can often actually reflect the age at diagnosis and as such will inevitably have limited precision in predicting the start of an individual's underlying biological pathology. Applying polygenic score analyses to the results of large sequencing studies of clinically well‐characterized cohorts will help overcome the inherent imprecision of measuring AAO and applying polygenic score analysis to PD score alleles that inevitably include a proportion of SNPs that are not causal.

Finally, we have used the term PD score allele, as this approach cannot differentiate the minority of true PD risk alleles from variants not associated with the disease. As such, the derived polygenic scores have little value for predicting an individual's risk of developing PD. However, measures of polygenic burden could prove useful in distinguishing PD patients whose disease liability is most likely to carry the largest or smallest genetic component. Identifying these individuals would benefit genetic recall studies and could facilitate a better understanding of how gene–gene and gene–environment interactions increase risk to PD.

## Authorship

N.M.W., V.E.‐P., and H.R.M. designed the overall study. N.W.W., T.G., A.B., P.H., and J.H. oversaw sample collection and recruitment and supervised all clinical aspects. V.E.‐P. performed all statistical analysis. N.M.W., V.E.‐P., H.R.M., S.L., M.A.N., and A.B.S. interpreted the results. All authors contributed to writing and editing the manuscript.

## Potential Conflicts of Interest

H.R.M.: grants, Welsh Assembly Government, Ipsen Fund, MNDA, PSP Association; speaking fees, Teva, UCB; advisory board, Abbvie, Teva, Boerhing‐Ingelheim; webcast fees, GSK; travel expenses, Teva, Medtronic; coapplicant on a patent application related to C9ORF72—Method for diagnosing a neurodegenerative disease (PCT/GB2012/052140). J.H.: consultancy, Eisai Pharma; grant, Biogen Idec Pharma.

## IPDGC consortium members and affiliations:

Mike A Nalls (Laboratory of Neurogenetics, National Institute on Aging, National Institutes of Health, Bethesda, MD, USA), Vincent Plagnol (UCL Genetics Institute, London, UK), Dena G Hernandez (Laboratory of Neurogenetics, National Institute on Aging; and Department of Molecular Neuroscience, UCL Institute of Neurology, London, UK), Manu Sharma (Department for Neurodegenerative Diseases, Hertie Institute for Clinical Brain Research, University of Tübingen, and DZNE, German Center for Neurodegenerative Diseases, Tübingen, Germany), Una‐Marie Sheerin (Department of Molecular Neuroscience, UCL Institute of Neurology), Mohamad Saad (INSERM U563, CPTP, Toulouse, France; and Paul Sabatier University, Toulouse, France), Javier Simón‐Sánchez (Department of Clinical Genetics, Section of Medical Genomics, VU University Medical Centre, Amsterdam, Netherlands), Claudia Schulte (Department for Neurodegenerative Diseases, Hertie Institute for Clinical Brain Research), Suzanne Lesage (INSERM, UMR_S975 [formerly UMR_S679], Paris, France; Université Pierre et Marie Curie‐Paris, Centre de Recherche de l'Institut du Cerveau et de la Moelle épiniére, Paris, France; and CNRS, Paris, France), Sigurlaug Sveinbjörnsdóttir (Department of Neurology, Landspítali University Hospital, Reykjavík, Iceland; Department of Neurology, MEHT Broomfield Hospital, Chelmsford, Essex, UK; and Queen Mary College, University of London, London, UK), Sampath Arepalli (Laboratory of Neurogenetics, National Institute on Aging), Roger Barker (Department of Neurology, Addenbrooke's Hospital, University of Cambridge, Cambridge, UK), Yoav Ben‐Shlomo (School of Social and Community Medicine, University of Bristol), Henk W Berendse (Department of Neurology and Alzheimer Center, VU University Medical Center), Daniela Berg (Department for Neurodegenerative Diseases, Hertie Institute for Clinical Brain Research and DZNE, German Center for Neurodegenerative diseases), Kailash Bhatia (Department of Motor Neuroscience, UCL Institute of Neurology), Rob M A de Bie (Department of Neurology, Academic Medical Center, University of Amsterdam, Amsterdam, Netherlands), Alessandro Biffi (Center for Human Genetic Research and Department of Neurology, Massachusetts General Hospital, Boston, MA, USA; and Program in Medical and Population Genetics, Broad Institute, Cambridge, MA, USA), Bas Bloem (Department of Neurology, Radboud University Nijmegen Medical Centre, Nijmegen, Netherlands), Zoltan Bochdanovits (Department of Clinical Genetics, Section of Medical Genomics, VU University Medical Centre), Michael Bonin (Department of Medical Genetics, Institute of Human Genetics, University of Tübingen, Tübingen, Germany), Jose M Bras (Department of Molecular Neuroscience, UCL Institute of Neurology), Kathrin Brockmann (Department for Neurodegenerative Diseases, Hertie Institute for Clinical Brain Research and DZNE, German Center for Neurodegenerative diseases), Janet Brooks (Laboratory of Neurogenetics, National Institute on Aging), David J Burn (Newcastle University Clinical Ageing Research Unit, Campus for Ageing and Vitality, Newcastle upon Tyne, UK), Elisa Majounie (Laboratory of Neurogenetics, National Institute on Aging), Gavin Charlesworth (Department of Molecular Neuroscience, UCL Institute of Neurology), Honglei Chen (Epidemiology Branch, National Institute of Environmental Health Sciences, National Institutes of Health, NC, USA), Patrick F Chinnery (Neurology M4104, The Medical School, Framlington Place, Newcastle upon Tyne, UK), Sean Chong (Laboratory of Neurogenetics, National Institute on Aging), Carl E Clarke (School of Clinical and Experimental Medicine, University of Birmingham, Birmingham, UK; and Department of Neurology, City Hospital, Sandwell and West Birmingham Hospitals NHS Trust, Birmingham, UK), Mark R Cookson (Laboratory of Neurogenetics, National Institute on Aging), J Mark Cooper (Department of Clinical Neurosciences, UCL Institute of Neurology), Jean Christophe Corvol (INSERM, UMR_S975; Universitó Pierre et Marie Curie‐Paris; CNRS; and INSERM CIC‐9503, Hôpital Pitié‐Salpêtrière, Paris, France), Carl Counsell (University of Aberdeen, Division of Applied Health Sciences, Population Health Section, Aberdeen, UK), Philippe Damier (CHU Nantes, CIC0004, Service de Neurologie, Nantes, France), Jean‐François Dartigues (INSERM U897, Université Victor Segalen, Bordeaux, France), Panos Deloukas (Wellcome Trust Sanger Institute, Wellcome Trust Genome Campus, Cambridge, UK), Günther Deuschl (Klinik für Neurologie, Universitätsklinikum Schleswig‐Holstein, Campus Kiel, Christian‐Albrechts‐Universität Kiel, Kiel, Germany), David T Dexter (Parkinson's Disease Research Group, Faculty of Medicine, Imperial College London, London, UK), Karin D van Dijk (Department of Neurology and Alzheimer Center, VU University Medical Center), Allissa Dillman (Laboratory of Neurogenetics, National Institute on Aging), Frank Durif (Service de Neurologie, Hôpital Gabriel Montpied, Clermont‐Ferrand, France), Alexandra Dürr (INSERM, UMR_S975; Université Pierre et Marie Curie‐Paris; CNRS; and AP‐HP, Pitié‐Salpêtrière Hospital), Sarah Edkins (Wellcome Trust Sanger Institute), Jonathan R Evans (Cambridge Centre for Brain Repair, Cambridge, UK), Thomas Foltynie (UCL Institute of Neurology), Jing Dong (Epidemiology Branch, National Institute of Environmental Health Sciences), Michelle Gardner (Department of Molecular Neuroscience, UCL Institute of Neurology), J Raphael Gibbs (Laboratory of Neurogenetics, National Institute on Aging; and Department of Molecular Neuroscience, UCL Institute of Neurology), Alison Goate (Department of Psychiatry, Department of Neurology, Washington University School of Medicine, MI, USA), Emma Gray (Wellcome Trust Sanger Institute), Rita Guerreiro (Department of Molecular Neuroscience, UCL Institute of Neurology), Clare Harris (University of Aberdeen), Jacobus J van Hilten (Department of Neurology, Leiden University Medical Center, Leiden, Netherlands), Albert Hofman (Department of Epidemiology, Erasmus University Medical Center, Rotterdam, Netherlands), Albert Hollenbeck (AARP, Washington DC, USA), Janice Holton (Queen Square Brain Bank for Neurological Disorders, UCL Institute of Neurology), Michele Hu (Department of Clinical Neurology, John Radcliffe Hospital, Oxford, UK), Xuemei Huang (Departments of Neurology, Radiology, Neurosurgery, Pharmacology, Kinesiology, and Bioengineering, Pennsylvania State University– Milton S Hershey Medical Center, Hershey, PA, USA), Isabel Wurster (Department for Neurodegenerative Diseases, Hertie Institute for Clinical Brain Research and German Center for Neurodegenerative diseases), Walter Mätzler (Department for Neurodegenerative Diseases, Hertie Institute for Clinical Brain Research and German Center for Neurodegenerative diseases), Gavin Hudson (Neurology M4104, The Medical School, Newcastle upon Tyne, UK), Sarah E Hunt (Wellcome Trust Sanger Institute), Johanna Huttenlocher (deCODE genetics), Thomas Illig (Institute of Epidemiology, Helmholtz Zentrum München, German Research Centre for Environmental Health, Neuherberg, Germany), Pálmi V Jónsson (Department of Geriatrics, Landspítali University Hospital, Reykjavík, Iceland), Jean‐Charles Lambert (INSERM U744, Lille, France; and Institut Pasteur de Lille, Université de Lille Nord, Lille, France), Cordelia Langford (Cambridge Centre for Brain Repair), Andrew Lees (Queen Square Brain Bank for Neurological Disorders), Peter Lichtner (Institute of Human Genetics, Helmholtz Zentrum München, German Research Centre for Environmental Health, Neuherberg, Germany), Patricia Limousin (Institute of Neurology, Sobell Department, Unit of Functional Neurosurgery, London, UK), Grisel Lopez (Section on Molecular Neurogenetics, Medical Genetics Branch, NHGRI, National Institutes of Health), Delia Lorenz (Klinik für Neurologie, Universitätsklinikum Schleswig‐Holstein), Codrin Lungu (National Institutes of Health Parkinson Clinic, NINDS, National Institutes of Health), Alisdair McNeill (Department of Clinical Neurosciences, UCL Institute of Neurology), Catriona Moorby (School of Clinical and Experimental Medicine, University of Birmingham), Matthew Moore (Laboratory of Neurogenetics, National Institute on Aging), Huw R Morris (MRC Centre for Neuropsychiatric Genetics and Genomics, Cardiff University School of Medicine, Cardiff, UK), Karen E Morrison (School of Clinical and Experimental Medicine, University of Birmingham; and Neurosciences Department, Queen Elizabeth Hospital, University Hospitals Birmingham NHS Foundation Trust, Birmingham, UK), Ese Mudanohwo (Neurogenetics Unit, UCL Institute of Neurology and National Hospital for Neurology and Neurosurgery), Sean S O'Sullivan (Queen Square Brain Bank for Neurological Disorders), Justin Pearson (MRC Centre for Neuropsychiatric Genetics and Genomics), Joel S Perlmutter (Department of Neurology, Radiology, and Neurobiology at Washington University, St Louis), Hjörvar Pétursson (deCODE genetics; and Department of Medical Genetics, Institute of Human Genetics, University of Tübingen), Pierre Pollak (Service de Neurologie, CHU de Grenoble, Grenoble, France), Bart Post (Department of Neurology, Radboud University Nijmegen Medical Centre), Simon Potter (Wellcome Trust Sanger Institute), Bernard Ravina (Translational Neurology, Biogen Idec, MA, USA), Tamas Revesz (Queen Square Brain Bank for Neurological Disorders), Olaf Riess (Department of Medical Genetics, Institute of Human Genetics, University of Tübingen), Fernando Rivadeneira (Departments of Epidemiology and Internal Medicine, Erasmus University Medical Center), Patrizia Rizzu (Department of Clinical Genetics, Section of Medical Genomics, VU University Medical Centre), Mina Ryten (Department of Molecular Neuroscience, UCL Institute of Neurology), Stephen Sawcer (University of Cambridge, Department of Clinical Neurosciences, Addenbrooke's hospital, Cambridge, UK), Anthony Schapira (Department of Clinical Neurosciences, UCL Institute of Neurology), Hans Scheffer (Department of Human Genetics, Radboud University Nijmegen Medical Centre, Nijmegen, Netherlands), Karen Shaw (Queen Square Brain Bank for Neurological Disorders), Ira Shoulson (Department of Neurology, University of Rochester, Rochester, NY, USA), Ellen Sidransky (Section on Molecular Neurogenetics, Medical Genetics Branch, NHGRI), Colin Smith (Department of Pathology, University of Edinburgh, Edinburgh, UK), Chris CA Spencer (Wellcome Trust Centre for Human Genetics, Oxford, UK), Hreinn Stefánsson (deCODE genetics), Francesco Bettella (deCODE genetics), Joanna D Stockton (School of Clinical and Experimental Medicine), Amy Strange (Wellcome Trust Centre for Human Genetics), Kevin Talbot (University of Oxford, Department of Clinical Neurology, John Radcliffe Hospital, Oxford, UK), Carlie M Tanner (Clinical Research Department, The Parkinson's Institute and Clinical Center, Sunnyvale, CA, USA), Avazeh Tashakkori‐Ghanbaria (Wellcome Trust Sanger Institute), François Tison (Service de Neurologie, Hôpital Haut‐Lévêque, Pessac, France), Daniah Trabzuni (Department of Molecular Neuroscience, UCL Institute of Neurology), Bryan J Traynor (Laboratory of Neurogenetics, National Institute on Aging), André G Uitterlinden (Departments of Epidemiology and Internal Medicine, Erasmus University Medical Center), Daan Velseboer (Department of Neurology, Academic Medical Center), Marie Vidailhet (INSERM, UMR_S975, Université Pierre et Marie Curie‐Paris, CNRS, UMR 7225), Robert Walker (Department of Pathology, University of Edinburgh), Bart van de Warrenburg (Department of Neurology, Radboud University Nijmegen Medical Centre), Mirdhu Wickremaratchi (Department of Neurology, Cardiff University, Cardiff, UK), Nigel Williams (MRC Centre for Neuropsychiatric Genetics and Genomics), Caroline H Williams‐Gray (Department of Neurology, Addenbrooke's Hospital), Sophie Winder‐Rhodes (Department of Psychiatry and Medical Research Council and Wellcome Trust Behavioural and Clinical Neurosciences Institute, University of Cambridge), Kári Stefánsson (deCODE genetics), Maria Martinez (INSERM UMR 1043; and Paul Sabatier University), Nicholas W Wood (UCL Genetics Institute; and Department of Molecular Neuroscience, UCL Institute of Neurology), John Hardy (Department of Molecular Neuroscience, UCL Institute of Neurology), Peter Heutink (Department of Clinical Genetics, Section of Medical Genomics, VU University Medical Centre), Alexis Brice (INSERM, UMR_S975, Université Pierre et Marie Curie‐Paris, CNRS, UMR 7225, AP‐HP, Pitié‐Salpêtrière Hospital), Thomas Gasser (Department for Neurodegenerative Diseases, Hertie Institute for Clinical Brain Research, and DZNE, German Center for Neurodegenerative Diseases), Andrew B Singleton (Laboratory of Neurogenetics, National Institute on Aging).
